# Arsenic trioxide inhibits the proliferation of myeloma cell line through notch signaling pathway

**DOI:** 10.1186/1475-2867-13-25

**Published:** 2013-03-13

**Authors:** Jiasheng Hu, Xiao Huang, Xiuli Hong, Quanyi Lu, Xiongpeng Zhu

**Affiliations:** 1 Department of Haematology, Zhongshan Hospital of Xiamen University, Xiamen, Fujian 361004, China; 2 Department of Haematology, First Hospital of Quanzhou Affiliated Fujian Medical University, Quanzhou, Fujian, 36200, China

**Keywords:** Arsenic trioxide, Multiple myeloma, Notch signal, Gene expression

## Abstract

Arsenic Trioxide (ATO) has shown remarkable efficacy for the treatment of multiple myeloma (MM). However, the mechanism by which ATO exerts its inhibitory effect on the proliferation of myeloma cells remains to be clarified. We study the inhibitory effect of ATO at various concentrations on the proliferation of the myeloma cell line RPMI 8226 and discussed the molecular mechanism of ATO on myeloma cell line. Our results proved that ATO had a significant dose-dependent and time-dependent inhibitory effect on the expressions of the Notch receptor (Notch1) and Notch ligand (Jag2). Data from the real-time PCR assay showed that the mRNA expression levels of the *Jag2* gene and its downstream gene *Hes1* were both significantly down-regulated after the myeloma cells were treated with ATO while the expression of the tumor suppressor gene *PTEN* was up-regulated. These results elucidated the molecular mechanism underlying the ATO mediated inhibition of myeloma cell proliferation. This is the first report on the anti-myeloma activity in myeloma cells through inhibition of the Notch signaling pathway.

## Background

Multiple myeloma (MM) is a hematologic malignancy that results from clonal proliferation of plasma cells in the bone marrow. Although major advances have been made in the treatment of MM in the recent years, MM remains incurable mostly because of the development of drug resistance [1-5]. Therefore, new therapeutic strategies are needed to improve patient outcome.

Arsenic Trioxide (ATO) has been demonstrated the efficacy and safety treatment for acute promyelocytic leukemia (APL), Preclinical in vitro and in vivo studies showed also that ATO has antimyeloma effects both as a single agent and in combination with other antimyeloma agents [6-9], in patients with relapsed MM refractory, ATO combination therapies with melphalan, thalidomide, and bortezomib have shown promising results [10,11].

Currently, the effects mechanism of ATO has been studied extensively on myeloma, Hayashi reported ATO induces apoptosis of MM cells via caspase-9 and overcomes the protective effect of IL-6 in the BM milieu by inhibiting JAK-STAT survival signaling, ATO reduces tumor necrosis factor (TNF) a-induced adhesion to bone marrow stromal cells (BMSCs) and the resultant induced secretion of cytokines (IL-6 and VEGF) that promote MM cell growth, survival, and migration [12], Wu reported that ATO can mediated growth inhibition of myeloma cells through intrinsic signaling pathway activation [13], but the precise the mechanism is still unclear.

Notch signaling influences multiple processes that govern normal morphogenesis, apoptosis, and cellular proliferation. This signaling is initiated by binding of a Notch ligand to the extracellular domain of the Notch receptor. Notch ligands include Delta and Jagged (Jag), and 4 members belong to the Notch family of receptors (Notch1 to Notch4) [14,15]. There is ample evidence linking Notch and hematologic malignancies, including Hodgkin and non-Hodgkin lymphomas, subsets of acute myeloid leukemia, and B-cell chronic lymphoid leukemia [16-18]. Inhibition of Notch expression by antisense retrovirus or pharmacologic block of γ-secretase activity has a marked antineoplastic effect in Notch-expressing transformed cells in vitro and in xenograft models [19,20].

Previous studies have suggested that Jag2 induces cell cycling in confluent fibroblasts susceptible to density-dependent inhibition of cell division and therefore may contribute to neoplastic transformation, Jag2 induces the secretion of interleukin-6 (IL-6), vascular endothelial growth factor and insulin-like growth factor [21]. These results indicated an important role of Notch signaling (Jag2) in the survival and growth of myeloma cells. Thus, Jag2 might be a new therapeutic target for myeloma treatment.

Although ATO has been studied as potential anti-myeloma treatment, we don’t know whether ATO could inhibits the proliferation of myeloma cell through Notch signaling pathway, in this study we examined ATO exerts anti-myeloma effects involving in activity toward Notch signaling pathway, our discovery supported that ATO decreased activity of Notch signaling in myeloma cell line and which may provide a novel molecular basis and rationale for the use of ATO in MM treatment.

## Materials and methods

### Cells and reagents

Myeloma cell line RPMI8226 was bought from Shanghai cell bank of Chinese Academy of Sciences, Cells were cultured in RPMI 1640 medium (Sigma–Aldrich, St. Louis, MO, USA) supplemented with 10% heat-inactivated fetal bovine serum (FBS), 100 U/mL penicillin, 100 mg/mL streptomycin, and 2 mmol/L lglutamine at 37°C in humidified air containing 5% carbon dioxide. Culture medium was replaced every 3 days. ATO power was purchased from Sigma Chemical Company and stored at room temperature, ATO power was dissolved in 1.0 N NaOH and resulted in a 0.25 mM stock solution, the ATO solution was diluted in culture media just before use. All experiments were conducted with cells in logarithmic phase. For Western blot, mouse monoclonal antibodies against Notch-1 and Jag-2 (Santa Cruz, CA) the control cell line is lymphocytes from healthy volunteer. The experimental procedures were performed within the Xiamen University Medical Research Council guidelines and were approved by Zhongshan Hospital Ethics Committees. Patients provided informed consent according to the Declaration of Helsinki.

### MTT assay and flow cytometry

Cells were treated with various concentrations of ATO for 48 h.

3-[4,5-Dimethylthiazol-2-yl]-2,5-diphenyltetrazolium bromide (MTT) dye was added during the last 4 h of incubation. Insoluble formazan complexes were solubilized using dimethyl sulfoxide (DMSO), and absorbance was measured at 540 nm using a Benchmark Plus microplate spectrophotometer (Bio-Rad, Hercules, CA). Each measurement was performed in triplicate and repeated at least once.

Cell cycle analysis was performed using a Becton Dickinson FACS flow cytometer according to the methods described previously [22]. Apoptosis of myeloma cells was detected by Annexin annexin V-PE/7-AAD staining. A total of 10,000 events were acquired and analyzed using a Becton Dickinson FACS flow cytometer.

### Western blot analysis

Cell lysates and total protein concentration was measured with the BCA Protein Assay Kit (Pierce Biotechnology, Rockford IL,USA). Equal amounts of protein were subjected to SDS-PAGE and proteins were transferred to nitrocellulose membranes (GE Healthcare, USA). The membrane was blocked in PBS containing 5% non-fat milk and 0.1% Tween-20, washed twice in PBS, and incubated with primary antibody at room temperature for 2 hours, followed by incubation with secondary antibody at room temperature for 45 minutes. Afterward, the proteins of interest were visualized using ECL chemiluminescence system (Santa Cruz Biotechnology, USA).

### Real-time polymerase chain reaction

RPMI 8226 cells were maintained in suspension for the indicated periods of time, and total RNA was isolated using RNA prep pure Blood Kit (Tiangen Biotech, China). First-strand cDNA was synthesized with 1 μg of RNA using ReverTra Ace^®^ qPCR RT Kit (Toyobo Co. Ltd., Japan) and random primers according to the manufacturer’s protocol. Polymerase chain reaction (PCR) was performed with 2.5 μL of cDNA template using the FS Universal SYBR Green Master (Roche) and target gene assay mix containing human *Jag2*, *Hes1*, and *PTEN* sequence-specific primers. The PCR reaction system consisted of ROX 25 μL; forward primer, 0.25 μL; reverse primer, 0.25 μL; cDNA, 5 μL; and diethyl phosphorocyanidate (DEPC), 19.5 μL. The final volume of the reaction mixture was 50 μL. We performed amplification using an 18S endogenous control assay mix (Amersham Life Sciences) as a control. The PCR conditions were as follows: 1 cycle at 95°C for 10 min, denaturation at 95°C for 15 s, and annealing/extension at 60°C for 1 min for a total of 40 cycles. PCR was performed in triplicate for each sample. Cycle numbers obtained at the log-linear phase of the reaction were plotted against a standard curve prepared using serially diluted control samples. The primers were synthesized by Yingjun (China).

*Jag2* Forward: 5′-CGAGCGAGTGTCGCATGCCGG-3′

Reverse: 5′-TGTTGCCGTACTGGTCGCAGG-3′;

*Hes1* Forward: 5′-TGATTTTGGATGCTCTGAAGA AAGATA-3′

Reverse: 5′-GCTGCAGGTTCCGGAGGT-3′;

*PTEN* Forward: 5′-ACCATAACCCACCACAGC-3′

Reverse: 5′-CAGTTCGTCCCTTTCCAG-3′.

### Statistical analysis

All values are expressed as the mean ± standard deviation (SD). Statistical analyses were performed using one-way analysis of variance (ANOVA) performed using the SPSS statistical software. Probability values of P < 0.05 were considered statistically significant.

## Results

### ATO inhibits myeloma cell growth and induces apoptosis

**Figure 1:**
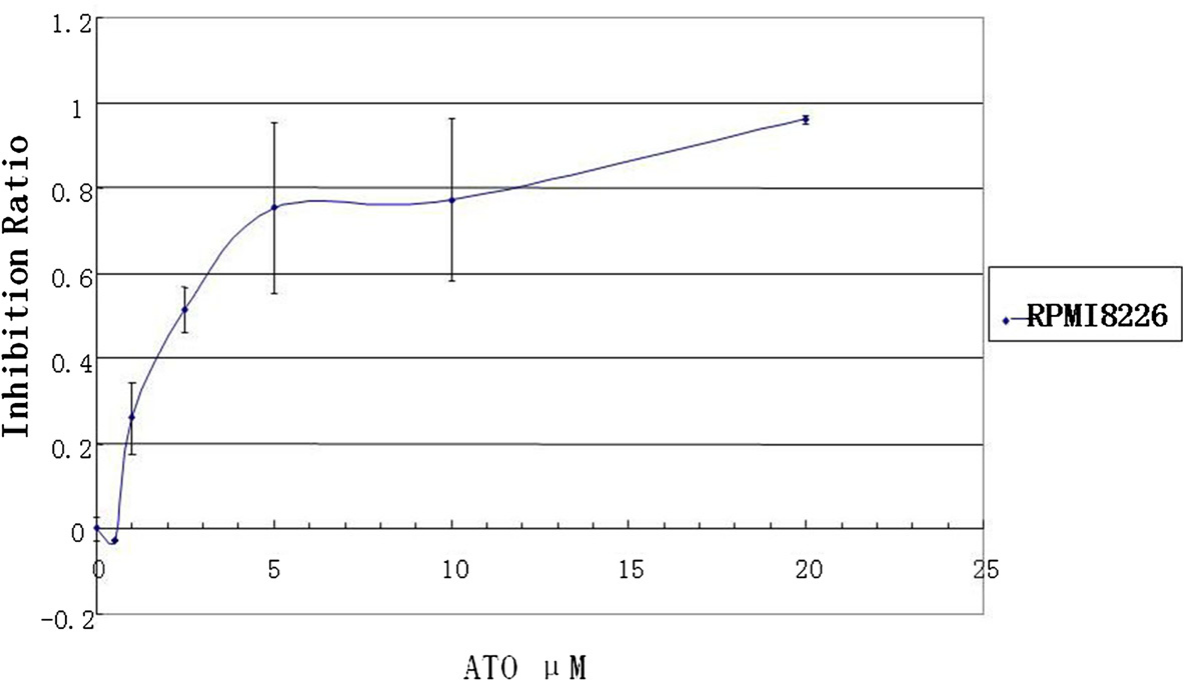
**ATO inhibits myeloma cells growth. **RPMI8226 cell was treated with ATO in different concentrations for 48 hours, cell growth inhibition was measured with the MTT as described in the Methods.

In order to determine the cell proliferation to ATO, RPMI8226 cell line was treated with drug at the concentration from 1 μM to 20 μM for 48 hours, ATO inhibited the growth of MM cells in a dose dependent manner (Figure 1). Fifty percent growth inhibition (IC50) in RPMI8226 cells at 48 hours was 2.4 μM ATO _,_ as determined by MTT assay, and this results demonstrate ATO inhibits the proliferation of MM cells at the relatively low concentration in cell line.

**Figure 2:**
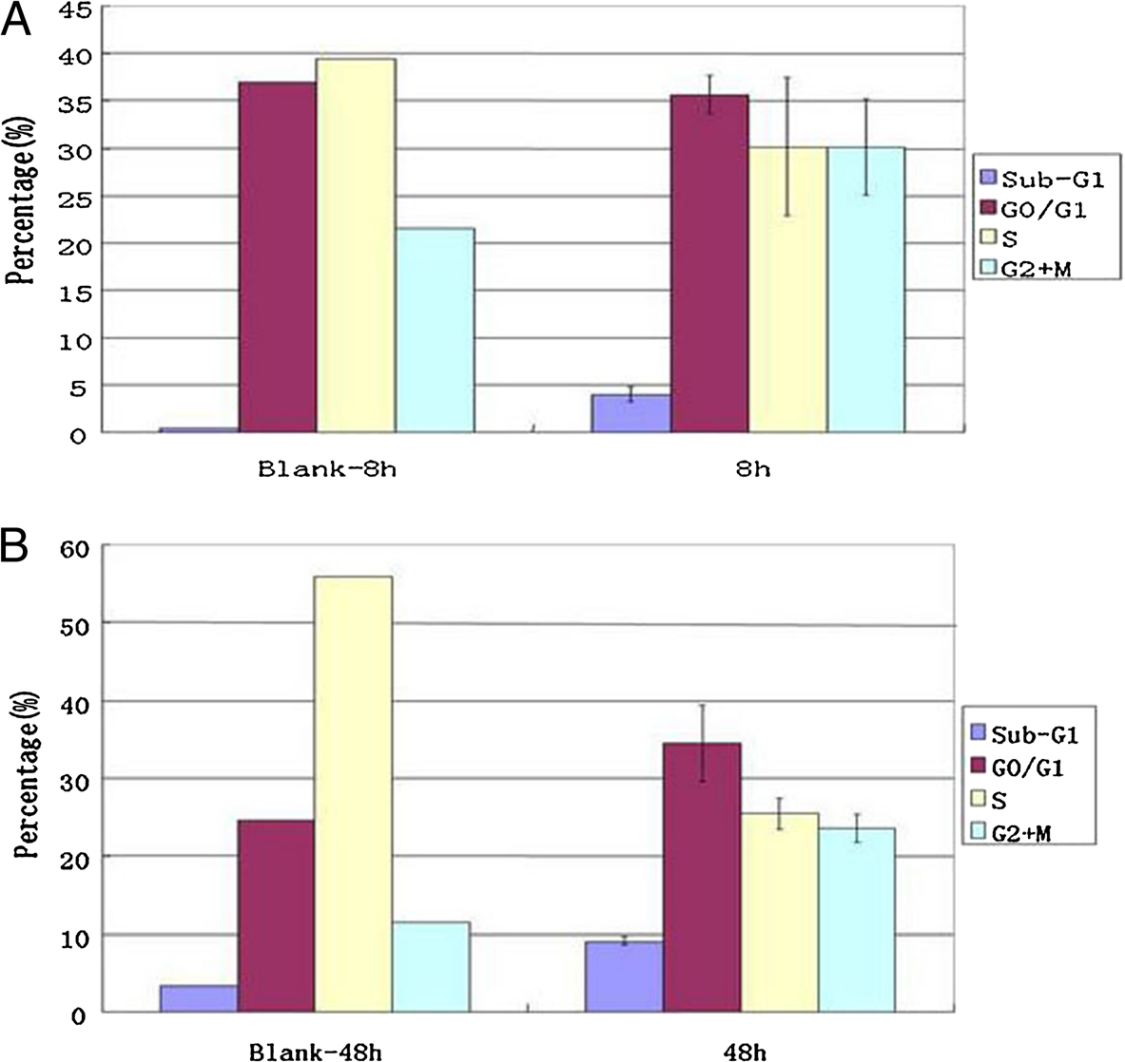
**ATO inhibits the growth and induces apoptosis of RPMI8226 cell. **The myeloma cells were cultured 8 and 48 hours with 5 μM ATO. The cell cycle and cell apoptosis were analysed by flow cytometry. Data are the mean + SD for three replicate measurements. * means statistical difference was observed between the treated group and control.

We also performed cell cycle analysis of RPMI 8226 cells treated with ATO (5 μM) by flow cytometry. The results showed that ATO treatment induced an increase in the number of cells in the G0/G1 phase, but induced a significant decrease in the number of cells in the S phase, and a sub-G1 peak appeared, which suggested apoptosis of the tumor cells (Figure 2).

Next, in order to confirm whether ATO decreased viability of tumor cells and induce apoptosis after treatment with ATO, Apoptosis of treated cells was measured by flow cytometry using annexin V-PE/7-AAD staining, these results demonstrated that ATO induce myeloma cell apoptosis in dose-dependent (Figure 2A).

### ATO inhibits Notch signaling levels in RPMI 8226 cells

**Figure 3:**
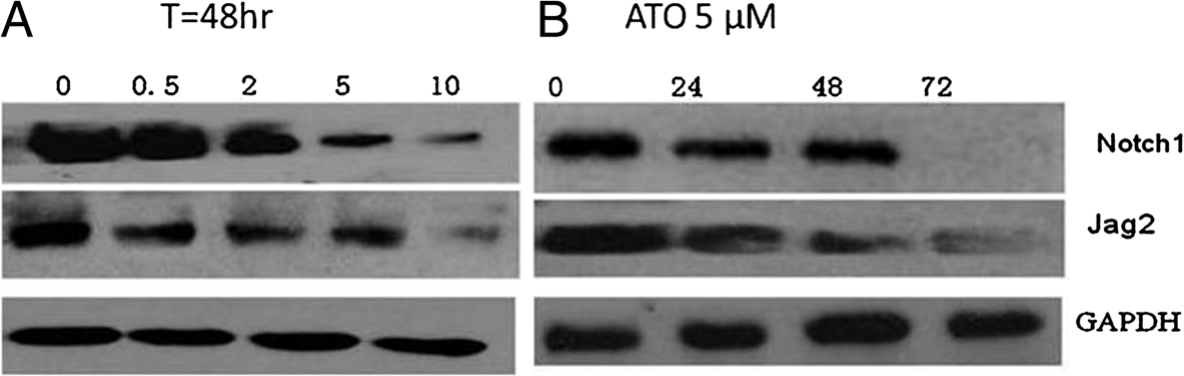
**ATO decreased the level of Notch signal expression of RPMI8226 cells, (A) myeloma cell was treated with different concentration ATO for 48 h, the expression of Notch1 and Jagged2 was obviously decreased. **(**B**) with 5 μM ATO, myeloma cell was treated for 24, 48, and 72 hours, the level of Notch1 ,jagged2 expression were significantly reduced.

To investigate whether a decrease in notch signaling level could be achieved by ATO treatment in the myeloma cells, RPMI 8226 cells was treated with ATO at various concentrations, the results showed a gradual decrease in the expression of Notch1 and Jag2 proteins after 48 h of incubation. Compared to the blank group, cells was treatment with 0.5, 2, 5, or 10 μM ATO showed a statistically significant reduction in the expression of Notch1 and Jag2 (*P* < 0.05). The reduction in protein expression was dose-dependent (Figure 3A). Furthermore, RPMI 8226 cells treated with 5 μM ATO for different durations (24, 48, or 72 h) showed a time-dependent reduction in the expression of Notch1 and Jag2 (Figure 3B).

**Figure 4:**
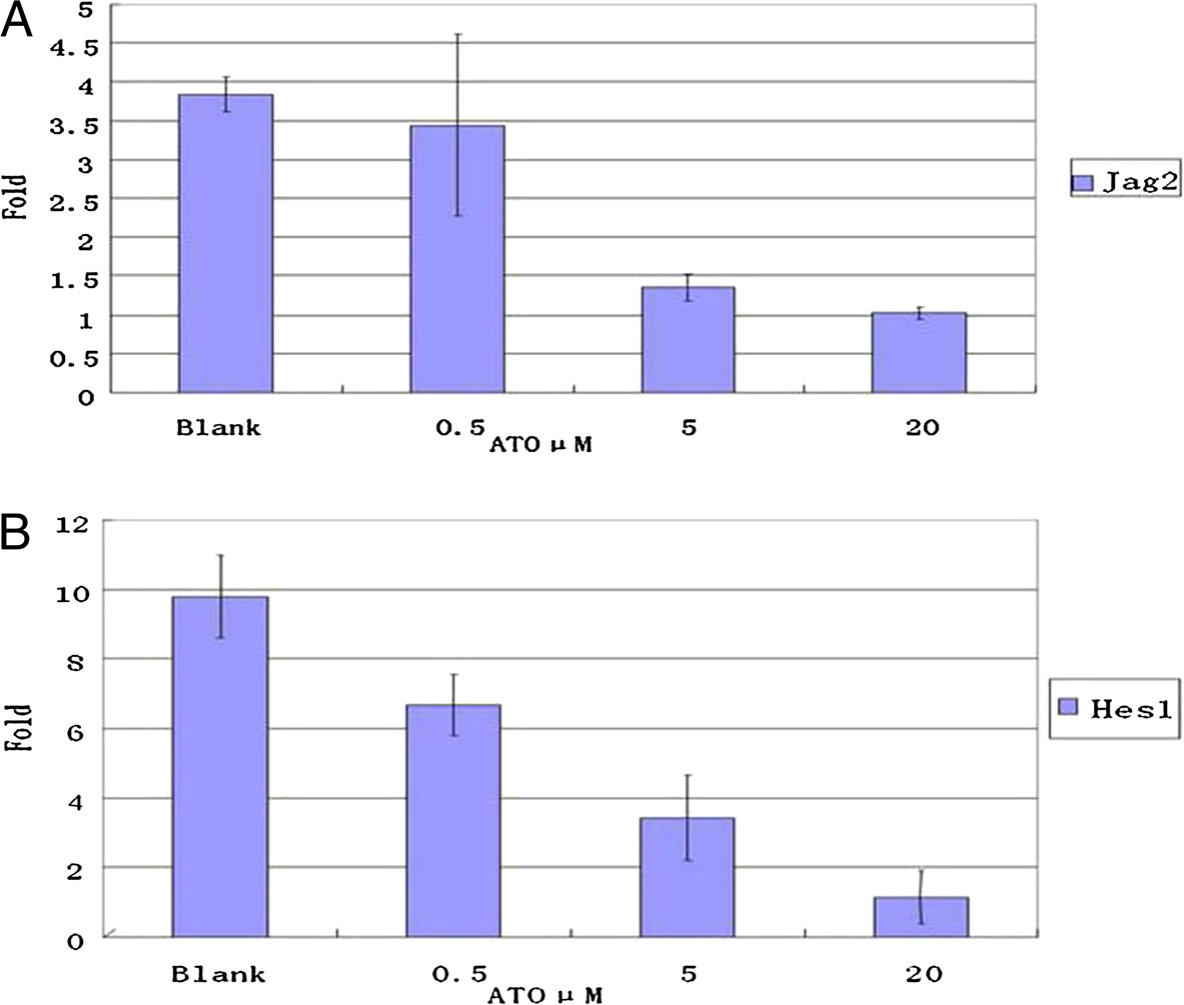
**ATO decreased the over expression of Notch signal in RPMI8226 cells detected by real time PCR. **With various concentration ATO(0.5, 5, and 20 μM), myeloma cell was treated for 24 hours, the over expression of Jagged2 and Hes1 was obviously decreased in RPMI8226 cell.

We also analyzed the effect of treatment with ATO for 24 h on Notch signaling in RPMI 8226 cells using real-time PCR. Our results showed that the expressions of the gene encoding the Notch signaling ligand Jag2 and its specific target gene *Hes1* decreased in RPMI 8226 cells, and the downregulation was dose-dependent. The difference in their expression levels was significant as analyzed by one-way ANOVA (*P* < 0.01, Figure 4).

### ATO up-regulate PTEN gene expression

**Figure 5:**
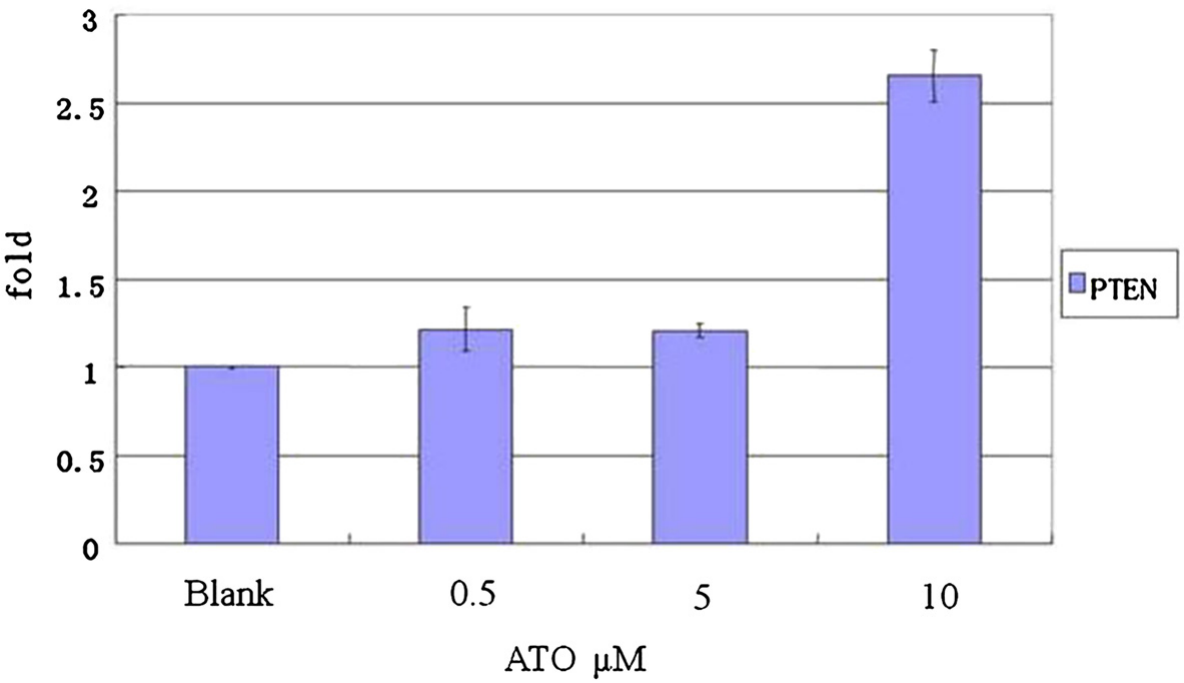
**ATO regulated the level of PTEN mRNA expression in RPMI8226. **Myeloma cell were treated with different concentrations ATO (0, 0.5, 5 and 10 μM) for 48 hours, PCR results showed that PTEN mRNA expression was increased in RPMI8226 cell.

We examined the effect of ATO treatment on changes in the expression levels of the *PTEN* gene in RPMI 8226 cells by using real-time PCR. Results showed that treatment with different concentrations of ATO for 48 h induced an up-regulation in *PTEN* gene expression, and the up-regulation was significantly different from that in the cells of the control group (*P* = 0.006). *PTEN* expression significantly increased after treatment with 10 μM ATO (Figure 5).

## Discussion

ATO has been used for refractory myeloma treatment in china, the precise mechanisms is uncertain, In the this study, We first demonstrate that ATO inhibits the proliferation of myeloma cells at low concentrations at time- and dose-dependent fashion, and Notch signal activity was decreased. This results are consistant with previous investigations [23]. In several leukemia and lymphoma cells, some studies have reported that the high concentrations of ATO treatment activated the Jun N-terminal kinase (JNK) and p38, members of stress-activated signal transduction pathways, and resulted in apoptosis [24,25]. But the relationship between Notch signal and ATO has not been studied. Our results indicated that down regulation of Notch signal may be one of molecular mechanism and ATO emerged as a promising class of anti-cancer drugs in myeloma.

*PTEN* gene mutation, deletion, downregulation, or malfunction of the protein encoding *PTEN* have all been found in a variety of cancers. *PTEN*-encoding protein has lipid and protein phosphatase activity, and it can be phosphorylated as a substrate. PTEN exerts its tumor suppressor effect by inhibiting the PI3P/AKT pathway through its lipid phosphatase activity. Previous studies found that the drug resistance to Notch signal inhibitor in T lymphoid leukemia patients was associated with *PTEN* gene deletion or abnormal expression [26,27]. Further research showed that activation of Notch signaling enabled upregulation of *Hes1*, a Notch-target gene. It combine with PTEN promoter, which results in the deactivation of PTEN, and consequently, in reduction of PTEN expression and activation of PI3P/AKT pathway, which eventually leads to cancer cell proliferation and apoptosis evasion. We observed an inhibitory effect of ATO on Notch signaling and downregulation of Hes1 expression as well as subsequent upregulation of PTEN expression. These results suggest that in MM, Notch signaling probably affects PTEN in a manner similar to that in T lymphoid leukemia, Notch signaling is negatively correlated to PTEN expression.

In conclusion, this study showed that ATO exerts anti-myeloma effects by inhibiting Notch signal and resulting in up-regulation of PTEN expression. The results identify ATO as a potential treatment for MM patients. Furthermore, they contribute to the understanding of the molecular mechanisms underlying the ATO-induced cell cycle arrest and apoptosis. Although a number of clinical studies have shown a moderate success of administering ATO to MM patients. More studies showed a synergic effect when ATO is administered in combination with other anti-MM drugs, such as bortezomib, the DNA methylation inhibitor 5-azacytidine and melphalan [28-30]. The results in the present study elaborated a novel molecular mechanism link among ATO and Notch signal, This is the first time to discover the relationship of ATO and Notch signal, our data here thus may provide an important insight into the molecular mechanism of anti-myeloma activity of ATO.

## Competing interests

The authors declare that they have no competing interests.

## Authors’ contributions

Dr JH performed experiments and drafted the paper, Dr QL organized the research plan and modified the manuscript, Dr XH, XH coordinated the study, participated in its design. All authors read and approved the final manuscript.
